# Frailty Change Scores Over Long-Term Follow Up in Patients With Coronary Artery Disease

**DOI:** 10.1093/geroni/igaa057.566

**Published:** 2020-12-16

**Authors:** Shahyar Gharacholou, Carolyn Flock, Ryan Lennon, Joshua Slusser, Leslie Cooper, Mandeep Singh

**Affiliations:** 1 Mayo Clinic, Jacksonville, Florida, United States; 2 Mayo Clinic, Rochester, Minnesota, United States

## Abstract

Background: Frailty assessment has often been performed only at baseline in cohort studies. Little is known regarding factors associated with changes in frailty indices over follow up in patients with coronary artery disease (CAD). Methods: Between 11/2008 - 8/2012, 142 community-dwelling adults ≥65 years of age with prior history of CAD (angina or revascularization) participated in a study of frailty assessment at Mayo Clinic Health System in La Crosse, WI. A sample of participants (n=45) were included for frailty re-assessment using the Fried frailty criteria approximately 5 years after their baseline measures. Frailty classification was based on absence of deficits (non-frail), 1-2 deficits (intermediately frail), or 3 or more deficits (frail). Factors associated with a change in frailty indices were studied. Results: There were 45 patients that had a second assessment of frailty indices. At baseline, 24 patients (60%) were not frail while 16 patients (40%) had at least 1 frail feature. At follow up, 20 patients (50%) were not frail while 20 patients (50%) had a frail feature. Those improving were more often being married, had prior revascularization, and were without angina. Interval development of slower gait speed (r = 0.46; p=0.004) and decreased grip strength (r = -0.39; p = 0.01) were associated with worsening frailty. Conclusions: Older adults with CAD are not often frail by standard criteria; however, incident deficits develop during long term follow-up. Spousal support, absence of angina, and change in functional indices are less often associated with frailty features.

